# Inherited kidney disease and CAKUT are common causes of kidney failure: ERA Registry Figure of the month

**DOI:** 10.1093/ckj/sfaf163

**Published:** 2025-05-30

**Authors:** Vianda S Stel, Alberto Ortiz, Anneke Kramer

**Affiliations:** ERA Registry, Department of Medical Informatics, Amsterdam UMC—Location University of Amsterdam, Amsterdam, the Netherlands; Amsterdam Public Health Research Institute, Quality of Care, Amsterdam, the Netherlands; Department of Nephrology and Hypertension, IIS-Fundacion Jimenez Diaz UAM, Madrid, Spain; Department of Medicine, Universidad Autonoma de Madrid, Madrid, Spain; ERA Registry, Department of Medical Informatics, Amsterdam UMC—Location University of Amsterdam, Amsterdam, the Netherlands; Amsterdam Public Health Research Institute, Quality of Care, wAmsterdam, the Netherlands

**Figure 1:**
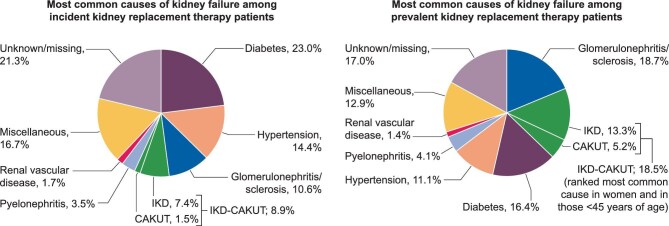
The distribution of causes of kidney failure among incident and prevalent kidney replacement therapy patients in 2019 in Europe. **Source:** Ortiz et al. CKJ 2024, https://doi.org/10.1093/ndt/gfae240, Fig. 1A and 3A. The figure was slightly adapted from the original figures (with PRDs ranked by frequency). **Explanation:** In 2019, IKD-CAKUT was the fourth most common cause of kidney failure among incident kidney replacement therapy patients (8.9%), following diabetes (23.0%), hypertension (14.4%) and glomerulonephritis (10.6%). Among prevalent kidney replacement therapy patients IKD-CAKUT (18.5%) and glomerulonephritis (18.7%) were the two most common causes of kidney failure. In prevalent women and those < 45 years of age IKD-CAKUT was ranked as most common cause. These findings stress the need for access to genetic testing in routine clinical practice to support optimal diagnosis, counselling and treatment of patients and their families.

